# Investigation of the relationship between chronic hepatitis B and tuberculosis using bioinformatics and systems biology approaches

**DOI:** 10.3389/fmed.2025.1519216

**Published:** 2025-06-12

**Authors:** Jinyi He, Xinyi Zhou, Danning Zhang, Yifei Cai, Hongyuan Pan, Tengda Huang, Fang He

**Affiliations:** ^1^Center of Infectious Diseases, West China Hospital, Sichuan University, Chengdu, China; ^2^Division of Liver Surgery, Department of General Surgery and Laboratory of Liver Surgery, and State Key Laboratory of Biotherapy, West China Hospital, Sichuan University, Chengdu, China; ^3^The College of Life Sciences, Sichuan University, Chengdu, China

**Keywords:** hepatitis B virus, tuberculosis, differentially expressed genes, protein–protein network (PPI), hub gene, drug molecule

## Abstract

**Background:**

Hepatitis B virus (HBV) is a globally prevalent pathogen that poses significant public health challenges. Active HBV replication can trigger immune responses that result in liver damage. Tuberculosis (TB), caused by *Mycobacterium tuberculosis* (*Mtb*), remains one of the leading causes of death from a single infectious agent worldwide. Notably, in TB patients with HBV infection and, the incidence of adverse events is six times higher than in those with TB alone, and HBV infection increases the risk of latent TB. However, the relationship between HBV and TB have not been thoroughly investigated.

**Methods:**

To elucidate the relationship between HBV and TB, we performed an integrated bioinformatics analysis using expression profiling and RNA sequencing data from the GSE83148 and GSE126614 datasets. We identified differentially expressed genes (DEGs) associated with both diseases and analyzed shared biological pathways, key genes, transcriptional regulatory networks, and gene-disease associations. Furthermore, we predicted potential therapeutic agents targeting these shared molecular features.

**Results:**

A total of 35 overlapping DEGs were identified for in-depth analysis. Functional enrichment revealed that these genes are involved in both immune-related pathways and cellular metabolic regulation, underscoring their potential role in the progression of HBV and TB. Protein–protein interaction (PPI) network analysis highlighted four hub genes: CCL2, CD69, EGR2, and CCL20. Additionally, 35 transcription factors (TFs) were predicted to regulate these hub genes. Several candidate drugs, including etoposide, 8-azaguanine, menaquinone, emetine and N-acetyl-L-cysteine, were identified as potential therapeutic options. The DEGs were also significantly associated with other conditions such as pneumonia.

**Conclusion:**

This study provides novel insights into the relationship between HBV and TB, offering potential targets for diagnosis and treatment. Our findings may contribute to the development of integrated strategies to manage HBV infection and TB more effectively.

## 1 Introduction

Chronic hepatitis B (CHB) caused by the hepatitis B virus (HBV) is a liver disease (1). HBV is one of the most common human pathogens and leads to serious public health problems. According to the World Health Organization’s (WHO) report in 2024, approximately 254 million people worldwide suffer from chronic hepatitis B (CHB), with an annual increase of approximately 1.2 million new infections (2). Active HBV replication can lead to immune liver injury, cirrhosis, hepatocellular carcinoma, and even liver failure (3). The tolerance effects of the hepatic environment and continued exposure of T cells to high antigen loads result in the suppression of T cell function in chronic HBV infection. This leads to the induction of HBV-specific T cell responses that are prompt and efficient in acute self-limiting infections but profoundly depleted in CHB (4).

Tuberculosis (TB) represents a significant global public health concern, caused by *Mycobacterium tuberculosis* (*Mtb*), with over 80% of cases and deaths occurring in low-income and middle-income countries (5). Tuberculosis can affect individuals of any age and gender, with a particularly high prevalence among adult males, but by 2022, only approximately two-fifths of individuals with drug-resistant tuberculosis will have access to treatment (5, 6). Tuberculosis is primarily a lung disease, with pulmonary tuberculosis accounting for 70% of cases. However, 30% of patients will also develop extrapulmonary disease, with the bacillus spreading to other organs such as the lymph nodes, bones, and meninges (7). The increasing prevalence of drug-resistant *Mtb* is attributable to a number of factors, including inappropriate use of TB medications, incorrect prescription by healthcare providers, poor-quality medications, and premature cessation of treatment (8).

The relationship between HBV and TB is not yet clear, but some studies have revealed some potential links between the two in clinical manifestations, immune response and drug interaction. In the setting of HBV infection, the development of TB is associated with a poor prognosis and poor response to treatment in patients, with adverse outcomes occurring six times more often than in patients infected only with TB and two times more often than in those experiencing liver failure, largely due to the effects of systemic immunity (8, 9). Individuals infected with HBV are at an elevated risk of developing latent tuberculosis, and vice versa (10). The likelihood of mixed infections is highest among those born in countries with a high prevalence of both diseases, particularly among Asians (10). It is established that cirrhosis due to CHB infection represents a risk factor for extrapulmonary TB and there are numerous reports of specific manifestations of TB in patients with cirrhosis and TB (11). In chronic HBV infection, HBV inhibits the ability of natural killer (NK) cells to produce interferon gamma (IFN-γ) by mediating an environment of high levels of immunosuppressive cytokines, such as interleukin-10 (IL-10), secreted by CD4^+^ CD25^+^ regulatory T cells (Tregs) (12, 13). IFN-γ production is an important effector mechanism of CD4^+^ T cell-mediated protective immunity in patients with TB (14, 15). Concurrently, TB diminishes antigen presentation by immune cells in the liver by inducing apoptosis of immune cells, particularly CD4^+^ T cells, thereby elevating the likelihood of HBV infection (16, 17). In TB patients with HBV infection and, liver dysfunction is exacerbated by the hepatotoxicity of anti-TB drugs and immune hepatic injury due to HBV replication (8). It has been asserted that the incidence of drug-induced liver injury (DILI) in TB patients with HBV infection is twice as high as the incidence in patients with TB alone (18). The results of these studies indicate a significant interaction between HBV infection and TB.

**Figure 1:**
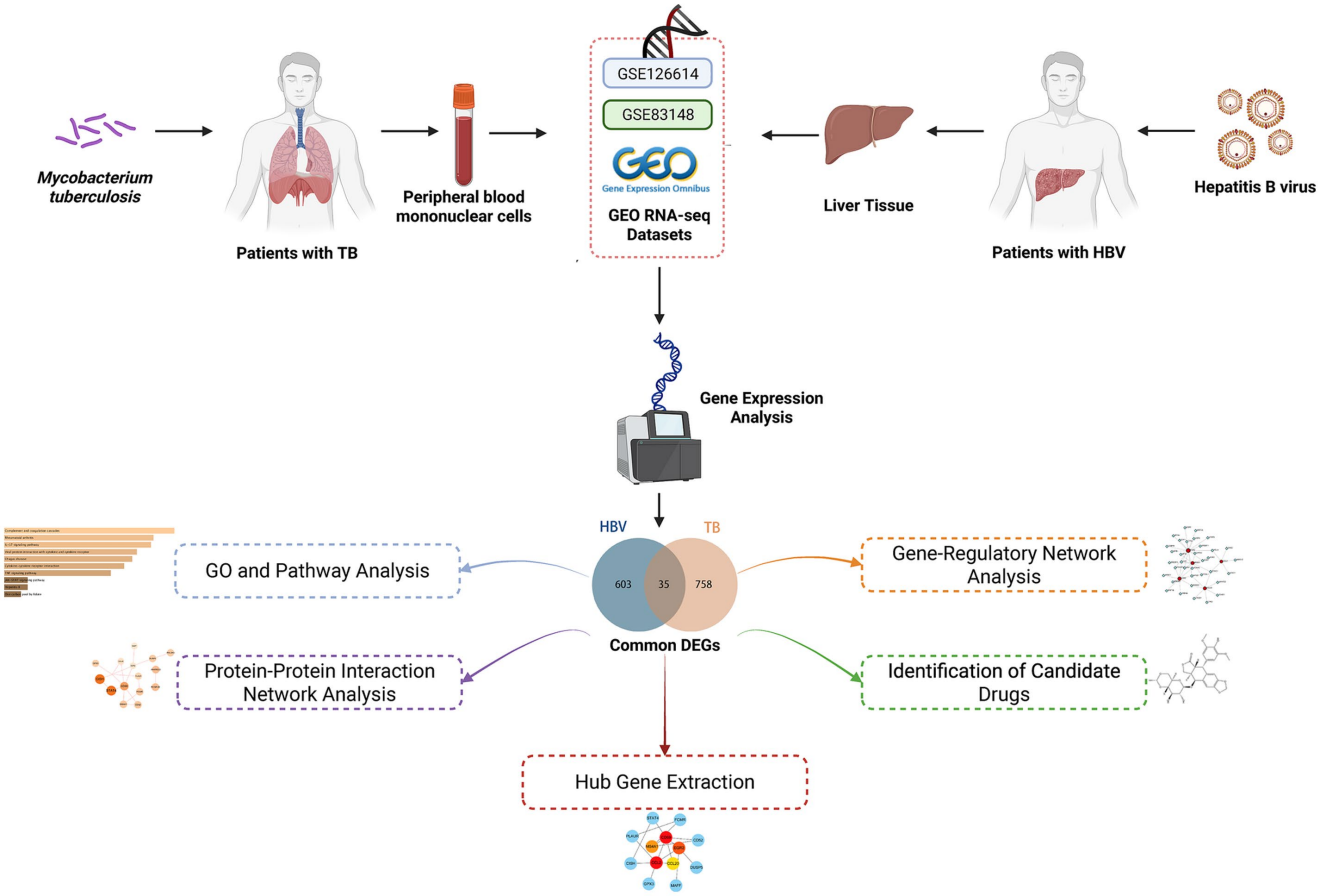
Schematic illustration of the overall general work flow of this study.

This study used the Gene Expression Omnibus (GEO) database (GSE83148 and GSE126614), systematically identifying the differentially expressed genes (DEGs) of the two diseases and focusing on their overlap. Based on common genes, in-depth pathway and gene function annotation analyses were performed. To narrow the focus of candidate genes, we constructed a protein–protein interaction (PPI) network of shared genes and identified hub genes in the network. Hub genes were used to explore the associated transcription factor (TF) regulatory network and predict potential therapeutic agents. Finally, we analyzed the association of these interacting genes with multiple disease contexts. Figure 1 illustrates the workflow of the analysis. This study provides insights into the molecular commonalities and response mechanisms of HBV and TB infection, intending to uncover new drug candidates and precise targets for HBV infection therapy to optimize patient prognosis.

## 2 Methods

### 2.1 Data acquisition

In order to ascertain the common pathogenetic processes shared by HBV and TB, we conducted a query of the RNA-seq datasets stored in the GEO databases of the National Center for Biotechnology Information (NCBI, https://www.ncbi.nlm.nih.gov/geo/) (19). The GSE83148 dataset (20) includes expression profiling by array data from 122 liver tissue samples with HBV infection and six healthy controls, generated using the Affymetrix Human Genome U133 Plus 2.0 Array platform based on the *Homo sapiens* reference genome. Statistical and detailed information on HBV patients, including age, gender, three clinical parameters (serum ALT, AST, and HBV-DNA), and inflammation grades of CHB, are provided in Supplementary Table 2. The GSE84044 dataset comprises liver biopsy samples from 124 patients with chronic hepatitis B (21). Transcriptomic profiling was performed using the Affymetrix HG U133 Plus 2.0 microarray platform (*Homo sapiens*). Each sample was evaluated for fibrosis stage (Scheuer S) and inflammation grade (Scheuer G) based on the Scheuer scoring system, according to the severity of inflammation and fibrosis (22). Detailed patient information is provided in Supplementary Table 3. Additionally, the GEO accession number of the TB dataset obtained through high-throughput sequencing on the Illumina HiSeq 2000 system (*Homo sapiens*) was GSE126614 (23), which contains the transcriptomic profiles for peripheral blood mononuclear cells from 19 healthy controls and 20 patients with active TB infection (Supplementary Table 4).

### 2.2 Identification of DEGs and common DEGs in HBV and TB infection

In transcriptomic studies, identifying DEGs relies on statistically significant differences in gene expression levels between different experimental conditions (24). For the dataset GSE83148, DEG analysis was performed using GEO2R^1^ (25), an online tool based on the limma package that facilitates the comparison of gene expression across different sample groups. For GSE126614, the DESeq2 package (26) in R was employed to identify DEGs. DESeq2 is a robust statistical method designed for differential expression analysis of count-based RNA-seq data. It utilizes shrinkage estimators for fold change and dispersion to improve the accuracy and reproducibility of the results. In both datasets, genes with an unadjusted *p*-value < 0.05 and |log₂ fold change| >1 were considered statistically significant. While false discovery rate (FDR) correction was not applied during initial screening, key findings were validated using independent datasets, and the results remained consistent. To explore the overlap of DEGs between HBV and TB, a Venn diagram analysis was conducted using the Jvenn online tool^2^ (27).

### 2.3 Gene ontology and pathway enrichment analysis

Gene enrichment analysis is a method for the investigation of gene expression data. Enrichment, in this context, signifies the categorization of genes in accordance with genomic annotation data (28). EnrichR is an interactive and collaborative gene list enrichment analysis tool that enables ontology function enrichment analysis and the classification of genes into the biological process (BP), cellular component (CC), and molecular function (MF) for GO analysis and signaling pathway enrichment (29). Four major databases were employed for gene enrichment analysis, including Reactome, WikiPathways, and the Kyoto Encyclopedia of Genes and Genomes (KEGG).

### 2.4 PPI network analysis

The STRING database (version 11.5) of protein interactions was employed to construct a PPI network of common DEGs, which was used to describe the physical and functional relationship between HBV and TB infection (30). A mean confidence score of 0.400 was employed in the analysis to construct the PPI network. Subsequently, Cytoscape (version 3.9.1) was employed for the visualization and further experimental investigation of the PPI network (31).

### 2.5 Hub gene extraction

The PPI network encompasses edges, nodes, and their interconnections. In this network, the most prominent nodes are assumed to be hub genes. The network was subsequently visualized using the Cytoscape platform. The hub genes were identified through the use of the Cytohubba plug-in^3^ in Cytoscape, which facilitated the analysis of nodes and the relationships between them (32). To ascertain the top four hub genes from the PPI network, the maximal clique centrality (MCC) function of Cytohubba was employed.

### 2.6 Gene-regulatory network analysis

Transcription factors are a class of protein factors with the capacity to recognise specific DNA sequences, thereby enabling the targeting and regulation of the efficiency and level of transcription of specific genes (33). To gain further insight, we employed NetworkAnalyst^4^ to delve more deeply into the JASPAR database, with the aim of identifying topologically plausible TFs that tend to bind to common DEGs (34). JASPAR is a regularly maintained, open-access database that stores human-edited, high-quality DNA-binding profiles of TFs in the form of position frequency matrices (PFMs) (35). Gene-TF interaction network was subsequently visualized using Cytoscape.

### 2.7 Identification of candidate drugs

The identification of potential therapeutic compounds targeting key disease-related genes was conducted using the EnrichR web platform and the Drug Signatures Database (DSigDB), which contains 22,527 gene expression profiles derived from drug-treated cells or tissues (36). The purpose of this analysis was to identify drugs that may modulate the expression of the hub genes identified from both HBV and TB datasets. Specifically, the hub genes were used as input into the EnrichR platform, which performed enrichment analysis based on the DSigDB database. This analysis compares the input gene set with known gene expression signatures induced by drug treatments, identifying compounds that are significantly associated with the input genes. EnrichR calculates enrichment scores and corresponding *p*-values to evaluate the statistical significance of these associations. Drugs with p-values less than 0.05 were considered to have significant potential to influence the expression of the hub genes. Based on these results, five candidate drugs were selected as potential therapeutic agents for further investigation.

## 3 Results

### 3.1 Identification of DEGs and shared DEGs between HBV and TB

**Figure 2:**
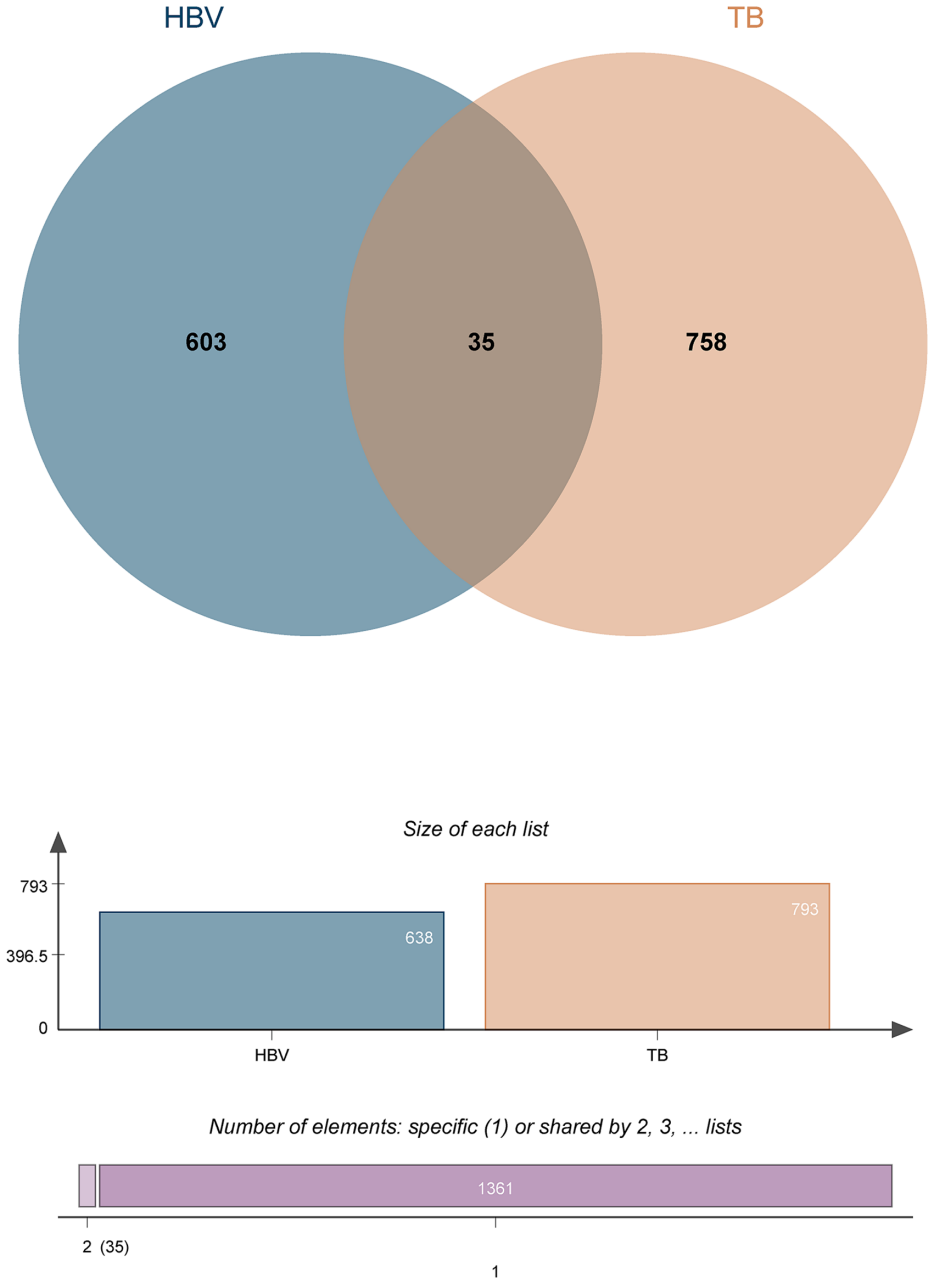
The study incorporates HBV (GSE83148) and TB (GSE126614). The Venn diagram revealed 35 common DEGs of HBV and TB.

DEGs from the transcriptional datasets were filled and the common DEGs causing HBV and TB infection were investigated to identify the common pathogenetic processes between HBV and TB infection. There are 638 DEGs, of which 582 DEGs were upregulated and 56 DEGs were downregulated, based on the RNA-seq profile of patients with HBV (GSE83148) (Supplementary Table 5). Likewise, we identified 793 DEGs, including 462 upregulated DEGs and 331 downregulated DEGs (Supplementary Table 6), from the evaluation of the TB dataset (GSE126614). The summarized information on the DEGs for HBV and TB infection is shown in Table 1. Furthermore, by performing the cross-comparison evaluation on Jvenn, 35 common DEGs were identified from the HBV and TB datasets (Figure 2). These findings reveal that the 35 shared genes identified in this study are involved in the regulation of HBV and TB infection, indicating that HBV and TB infection share some common mechanisms and pathogenic processes.

**Table 1:** Overview of the datasets in this analysis.

Disease name	GEO accession	GEO platform	Total DEGs count	Up-modulated DEGs count	Down-modulated DEGs count
HBV	GSE83148	GPL20301	638	582	56
TB	GSE126614	GPL11154	797	465	332

### 3.2 Analyses of gene ontology and pathway enrichment

**Figure 3:**
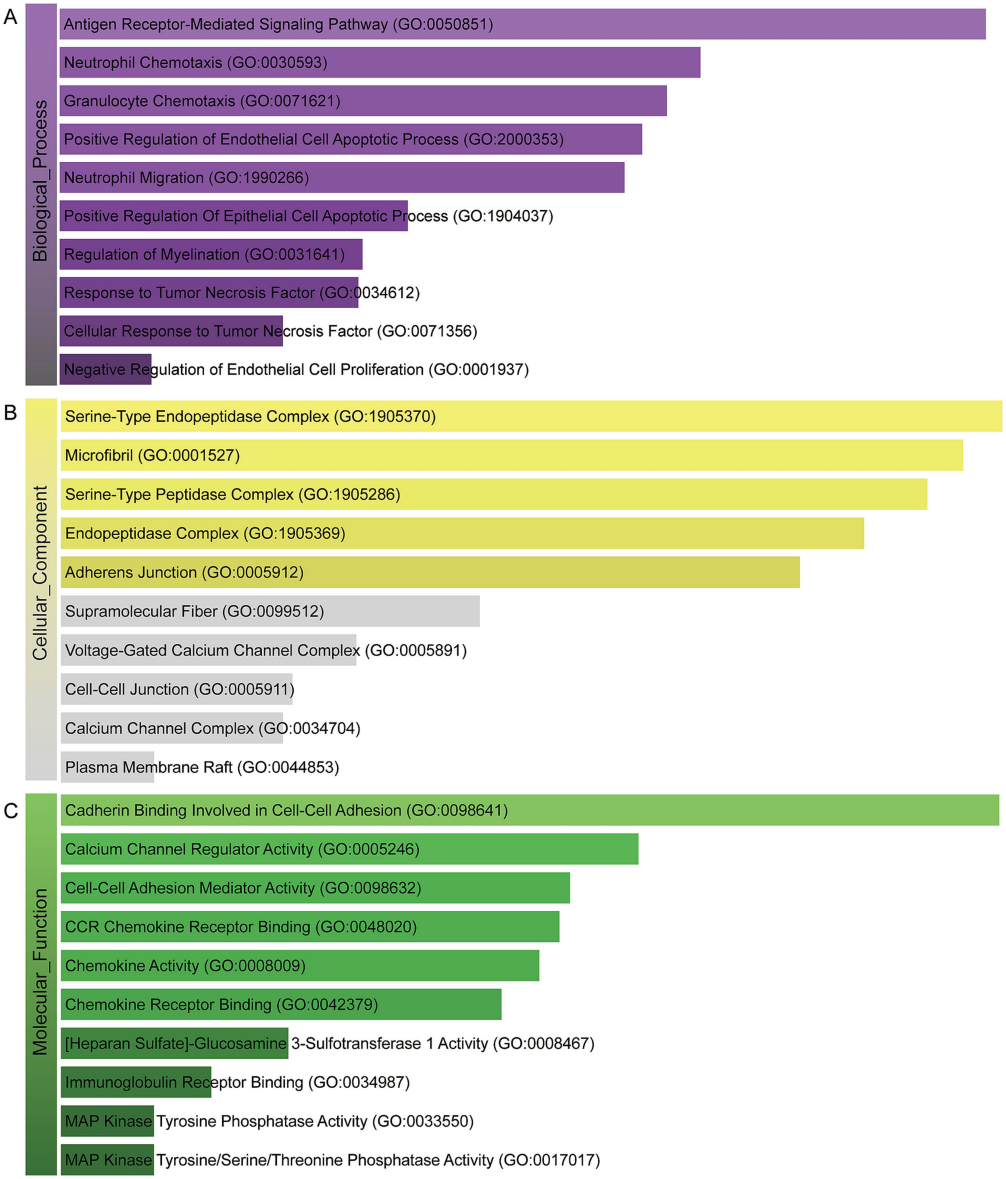
The bar chart of the GO assessment of the shared DEGs between HBV and TB. **(A)** Biological processes. **(B)** Cellular components. **(C)** Molecular function.

A deeper comprehension of the underlying biological implications and the prevalent signaling pathways within the dataset was sought through the utilization of the commonly DEGs. Here we implement GO enrichment methods and path enrichment methods through the EnrichR platform. Table 2 presents a summary of the top 10 terms related to the biological process, molecular function, and cellular component categories, as identified through the gene ontology analysis. As illustrated in Figure 3, the comprehensive ontology analysis is presented in the form of a bar graph for each category. It is noteworthy that there has been a pronounced elevation in immune-related pathways, encompassing Antigen Receptor-Mediated Signaling Pathway (GO:0050851), Neutrophil Chemotaxis (GO:0030593), Granulocyte Chemotaxis (GO:0071621), Neutrophil Migration (GO:1990266), Response to Tumor Necrosis Factor (GO:0034612) Cellular Response to Tumor Necrosis Factor (GO:0071356).

**Table 2:** Ontological analysis of common DEGs between HBV and TB.

Category	GO ID	Term	*p*-value	Genes
GO biological process	GO:0050851	Antigen receptor-mediated signaling pathway	8.59 × 10^−5^	IGHM/PDE4B/MS4A1/TNFRSF21
	GO:0030593	Neutrophil chemotaxis	2.48 × 10^−4^	CCL20/PDE4B/CCL2
	GO:0071621	Granulocyte chemotaxis	2.81 × 10^−4^	CCL20/PDE4B/CCL2
	GO:2000353	Positive regulation of endothelial cell apoptotic process	3.08 × 10^−4^	RGCC/CCL2
	GO:1990266	Neutrophil migration	3.29 × 10^−4^	CCL20/PDE4B/CCL2
	GO:1904037	Positive regulation of epithelial cell apoptotic process	7.36 × 10^−4^	RGCC/CCL2
	GO:0031641	Regulation of myelination	8.70 × 10^−4^	EGR2/TNFRSF21
	GO:0034612	Response to tumor necrosis factor	8.84 × 10^−4^	CCL20/CCL2/TNFRSF21
	GO:0071356	Cellular response to tumor necrosis factor	0.001	CCL20/CCL2/TNFRSF21
	GO:0001937	Negative regulation of endothelial cell proliferation	0.001	RGCC/CCL2
GO cellular component	GO:1905370	Serine-type endopeptidase complex	0.017	PLAUR
	GO:0001527	Microfibril	0.019	MFAP4
	GO:1905286	Serine-type peptidase complex	0.021	PLAUR
	GO:1905369	Endopeptidase complex	0.024	PLAUR
	GO:0005912	Adherens junction	0.028	CDC42EP1/S100A11
	GO:0099512	Supramolecular fiber	0.061	MFAP4
	GO:0005891	Voltage-gated calcium channel complex	0.082	PDE4B
	GO:0005911	Cell–cell junction	0.096	CDC42EP1/S100A11
	GO:0034704	Calcium channel complex	0.098	PDE4B
	GO:0044853	Plasma membrane raft	0.134	MS4A1
GO molecular function	GO:0098641	Cadherin binding involved in cell–cell adhesion	0.001	CDC42EP1/S100A11
	GO:0005246	Calcium channel regulator activity	0.002	SGK1/GEM
	GO:0098632	Cell–cell adhesion mediator activity	0.003	CDC42EP1/S100A11
	GO:0048020	CCR chemokine receptor binding	0.003	CCL20/CCL2
	GO:0008009	Chemokine activity	0.003	CCL20/CCL2
	GO:0042379	Chemokine receptor binding	0.003	CCL20/CCL2
	GO:0008467	[Heparan sulfate]-glucosamine 3-sulfotransferase 1 activity	0.009	HS3ST3B1
	GO:0034987	Immunoglobulin receptor binding	0.012	IGHM
	GO:0033550	MAP kinase tyrosine phosphatase activity	0.016	DUSP5
	GO:0017017	MAP kinase tyrosine/serine/threonine phosphatase activity	0.016	DUSP5

**Figure 4:**
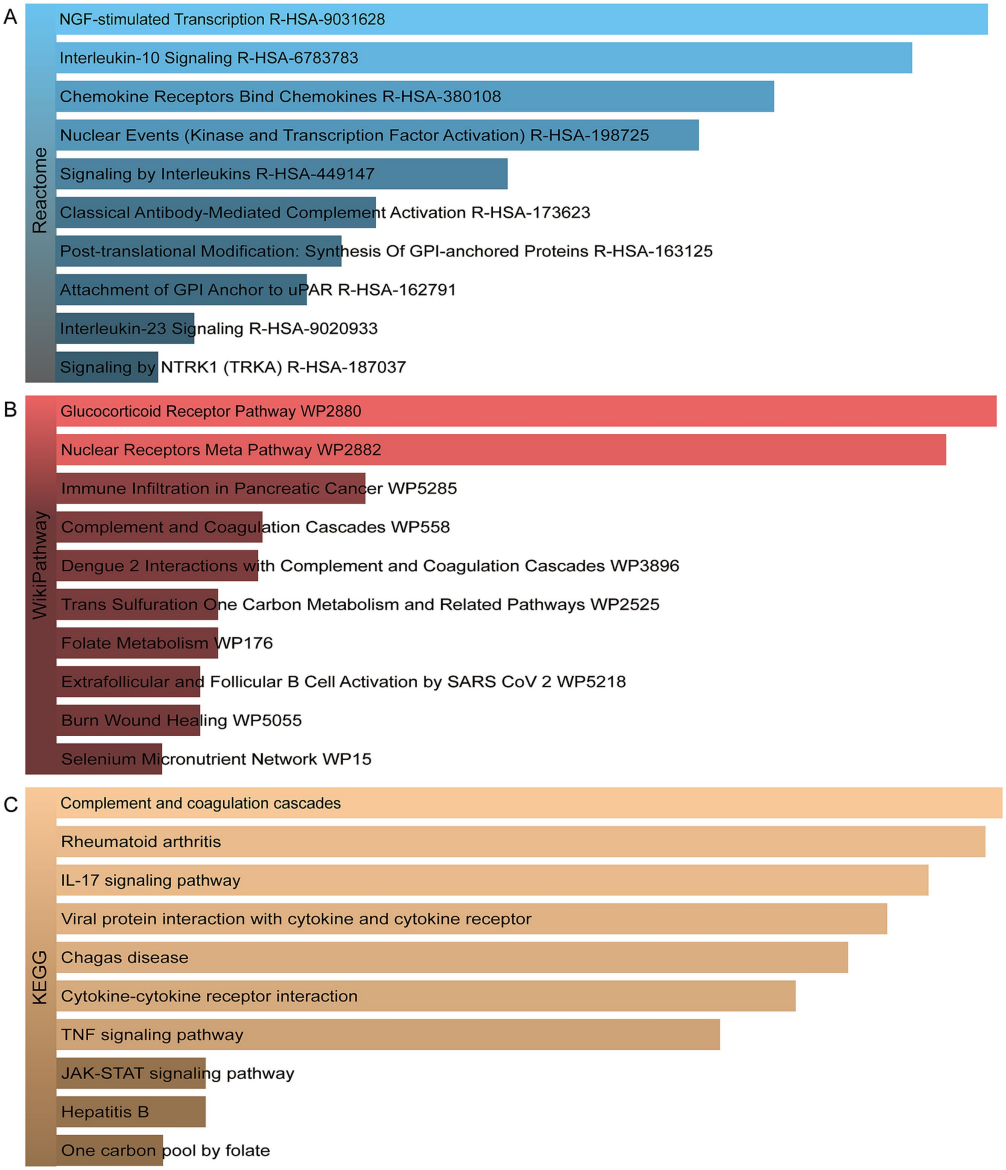
The bar graphs of the pathway enrichment of the shared DEGs between HBV and TB. **(A)** Reactome. **(B)** WikiPathways. **(C)** KEGG.

In order to identify the most prominent pathways associated with the mutual DEGs, three global databases were employed: Reactome, WikiPathways, and KEGG. A summary of the 10 most significant pathways identified across the three databases is provided in Table 3. Furthermore, the results of the pathway enrichment study are illustrated in the bar graph in Figure 4. The examination of the enriched pathways revealed that the majority of DEGs are associated with immune-related regulatory mechanisms, including Interleukin-10 Signaling R-HSA-6783783, Signaling By Interleukins R-HSA-449147, Classical Antibody-Mediated Complement Activation R-HSA-173623, Interleukin-23 Signaling R-HSA-9020933, Complement and Coagulation Cascades WP558, IL-17 signaling pathway, Viral protein interaction with cytokine and cytokine receptor, Cytokine-cytokine receptor interaction and TNF signaling pathway. Additionally, analysis of the data set using WikiPathways and KEGG demonstrated that the shared DEGs may play a role in the progression of various diseases. Notably HBV was included, as was SARS-CoV-2, a highly infectious disease along with HBV and TB. The DEGs were also significantly enriched in cell signaling and regulation of gene expression, such as NGF-stimulated Transcription R-HSA-9031628, Nuclear Events (Kinase and Transcription Factor Activation) R-HSA-198725, Signaling by NTRK1 (TRKA) R-HSA-187037 and JAK-STAT signaling pathway. These results clearly indicate that, through cellular metabolic regulation and immune-related pathways, these mutual DEGs are involved in the onset and development of HBV and TB infection.

**Table 3:** Pathway enrichment analysis of common DEGs between HBV and TB.

Category	Pathways	*p*-value	Genes
Reactome	NGF-stimulated transcription R-HSA-9031628	0.002	EGR2/SGK1
	Interleukin-10 signaling R-HSA-6783783	0.003	CCL20/CCL2
	Chemokine receptors bind chemokines R-HSA-380108	0.004	CCL20/CCL2
	Nuclear events (kinase and transcription factor activation) R-HSA-198725	0.005	EGR2/SGK1
	Signaling by interleukins R-HSA-449147	0.008	CISH/CCL20/STAT4/CCL2
	Classical antibody-mediated complement activation R-HSA-173623	0.010	C1QC
	Post-translational modification: synthesis of GPI-anchored proteins R-HSA-163125	0.011	CD52/PLAUR
	Attachment of GPI anchor to uPAR R-HSA-162791	0.012	PLAUR
	Interleukin-23 signaling R-HSA-9020933	0.016	STAT4
	Signaling by NTRK1 (TRKA) R-HSA-187037	0.017	EGR2/SGK1
WikiPathways	Glucocorticoid receptor pathway WP2880	0.001	CCL20/PDE4B/CCL2/SPRY1
	Nuclear receptors meta pathway WP2882	0.001	GPX3/CCL20/MAFF/PDE4B/CCL2/SPRY1
	Immune infiltration in pancreatic cancer WP5285	0.002	CCL20/CCL2
	Complement and coagulation cascades WP558	0.005	PLAUR/C1QC
	Dengue two interactions with complement and coagulation cascades WP3896	0.005	PLAUR/C1QC
	Trans sulfuration one carbon metabolism and related pathways WP2525	0.006	GPX3/TYMS
	Folate metabolism WP176	0.006	GPX3/CCL2
	Extrafollicular and follicular B cell activation by SARS-CoV-2 WP5218	0.007	CD69/MS4A1
	Burn wound healing WP5055	0.007	CCL2/S100A11
	Selenium micronutrient network WP15	0.010	GPX3/CCL2
KEGG	Complement and coagulation cascades	0.010	PLAUR/C1QC
	Rheumatoid arthritis	0.012	CCL20/CCL2
	IL-17 signaling pathway	0.012	CCL20/CCL2
	Viral protein interaction with cytokine and cytokine receptor	0.013	CCL20/CCL2
	Chagas disease	0.014	CCL2/C1QC
	Cytokine-cytokine receptor interaction	0.015	CCL20/CCL2/TNFRSF21
	TNF signaling pathway	0.016	CCL20/CCL2
	JAK-STAT signaling pathway	0.033	CISH/STAT4
	Hepatitis B	0.033	EGR2/STAT4
	One carbon pool by folate	0.034	TYMS

### 3.3 Protein–protein interaction analysis and hub gene extraction

**Figure 5:**
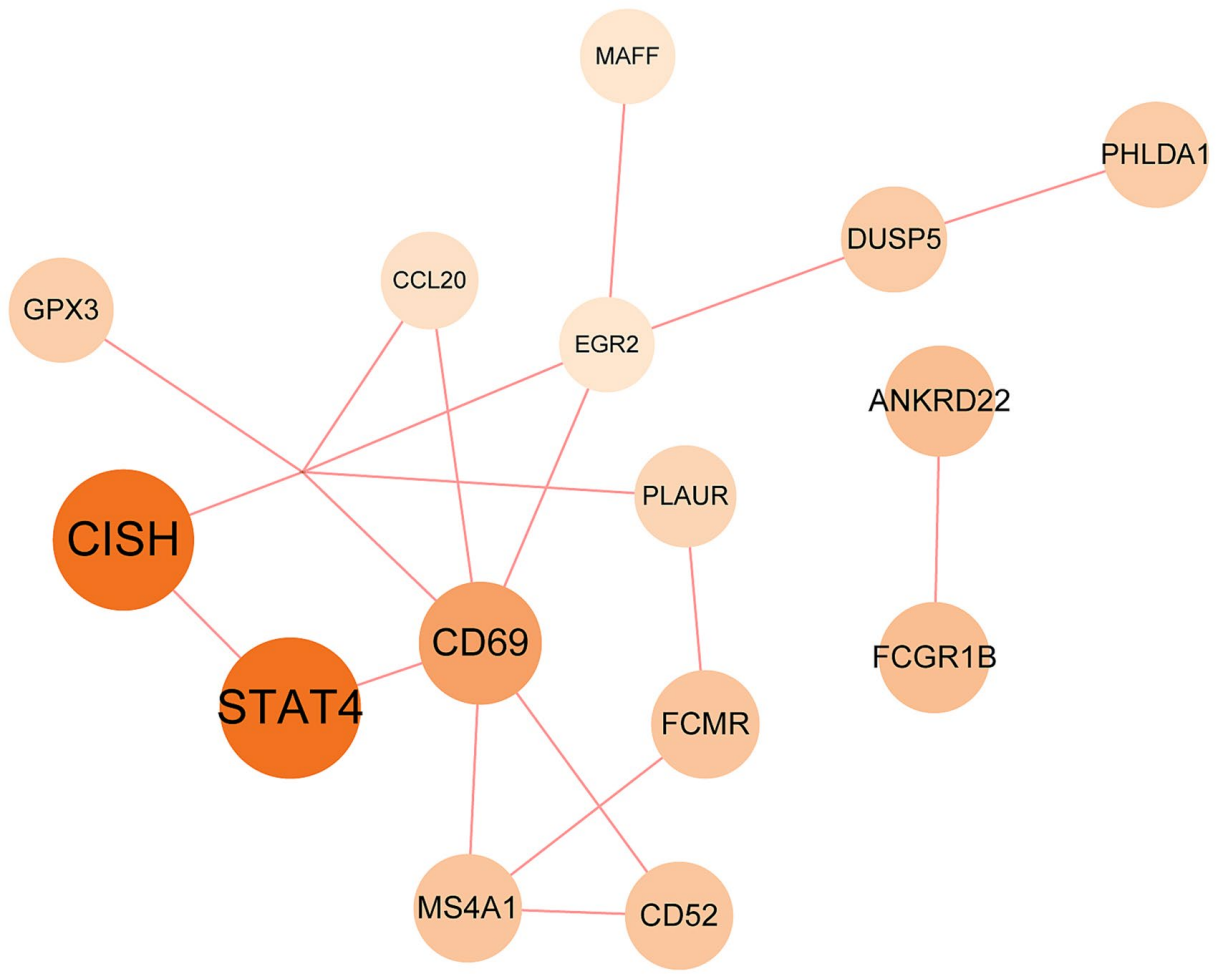
PPI network of the mutual DEGs between HBV and TB. The size and color depth of the circles represent the extent of protein intercorrelation. The most prominent nodes have been identified as hub genes. The nodes and the edges of the figure represent DEGs and the interactions between the nodes, respectively. The PPI network contains 15 edges and 18 nodes.

**Figure 6:**
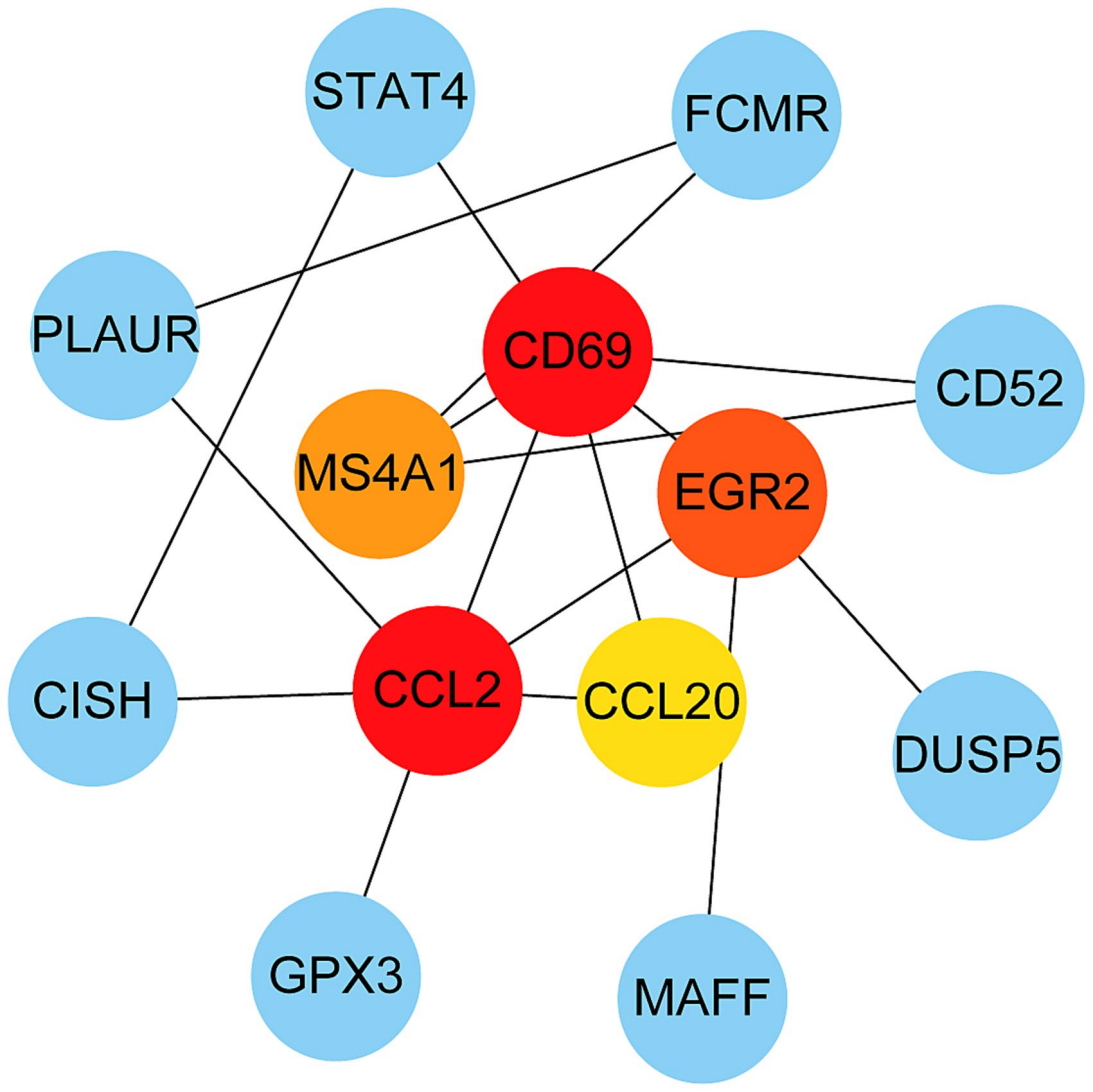
PPI network from all the shared DEGs is constructed by Cytohubba plugin in Cytosacpe. Red nodes present the selected top four hub genes. The network has 13 nodes and 17 edges.

**Figure 7:**
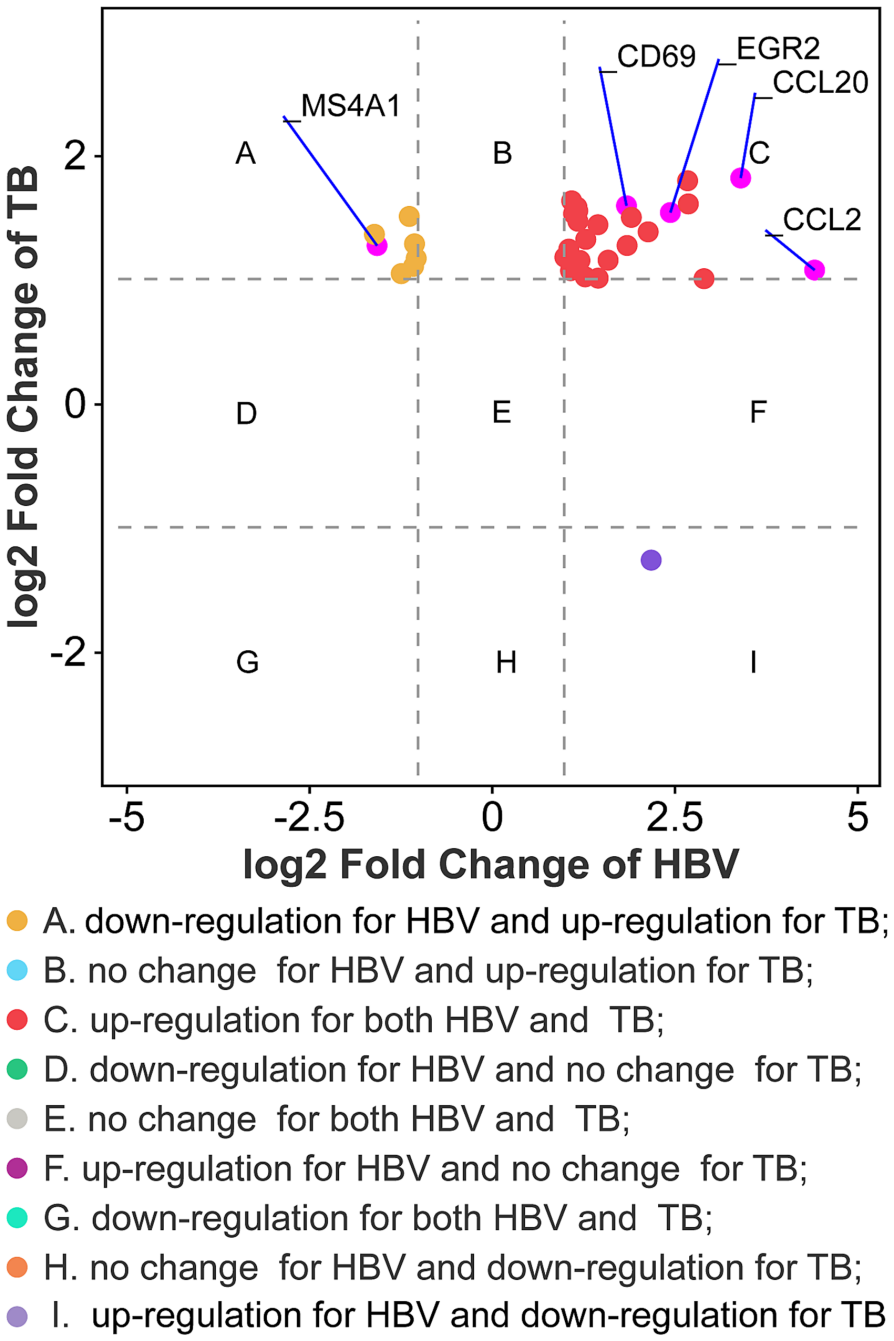
Fold changes of HBV and TB at transcriptional level. Nine categories in different colors indicate nine responsive groups (|log_2_ Fold Change| ≥1 and *p*-value <0.05).

**Figure 8:**
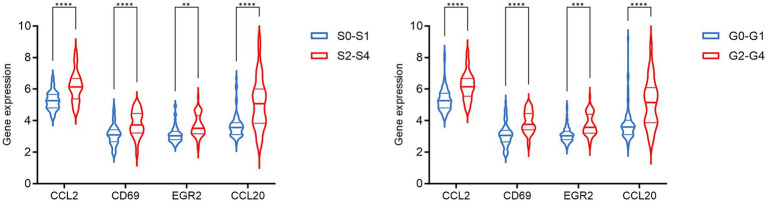
The expression level of hub genes in HBV patients in the GSE84044 dataset. Each sample was evaluated for fibrosis stage (Scheuer S) and inflammation grade (Scheuer G) based on the Scheuer scoring system, according to the severity of inflammation and fibrosis. ^**^p < 0.01, ^***^p < 0.001, and ^****^p < 0.0001.

The PPI network was constructed using the STRING database and visualized with Cytoscape, enabling the identification of interactions and adhesion-related pathways among the shared DEGs. The resulting PPI network, comprising 15 nodes and 19 edges, is shown in Figure 5. Using the CytoHubba plugin, five hub genes were identified from the network. Figure 6 presents the submodule network of candidate hub gene interactions, which contains 13 nodes and 17 edges. Based on the MCC algorithm, the top five candidate hub genes were identified as C-C motif chemokine ligand 2 (CCL2), CD69 molecule (CD69), early growth response protein 2 (EGR2), and C-C motif chemokine ligand 20 (CCL20) (Supplementary Table 7). As illustrated in Figure 7, we further examined the expression profiles of the shared DEGs in HBV and TB, with particular attention to the differential expression of these five candidate hub genes. Among them, four genes—*CCL2, CD69, EGR2,* and *CCL20*—were found to be consistently upregulated in both HBV and TB conditions. In addition, in order to ensure the repeatability of this study, we introduced an external data set (GSE84044) to verify the expression levels of four hub genes, as shown in Figure 8. Therefore, we focused subsequent analyses on these four genes, which may serve as potential biomarkers for the shared pathogenetic mechanisms of HBV and TB infections. These findings could also provide insights into the development of novel therapeutic strategies targeting both diseases.

### 3.4 Determination of regulatory networks at the transcriptional level

**Figure 9:**
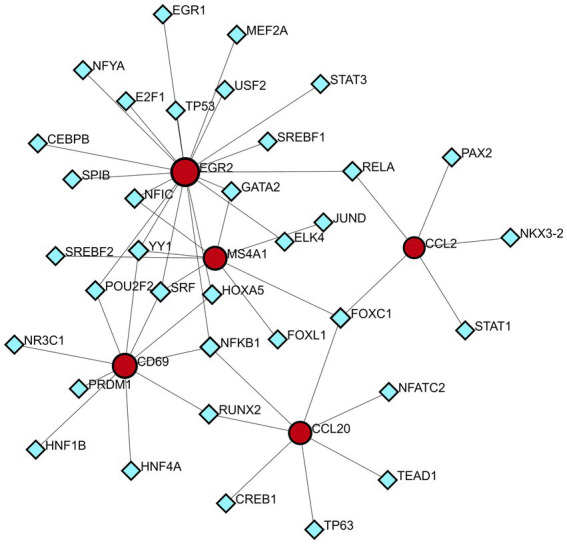
DEG-TFs interaction network created by the NetworkAnalyst. The red nodes represent gene symbols interacting with TFs while the blue nodes represent TFs.

A technology based on the network was used to decipher the regulatory TFs in order to explore the transcriptional regulation of the hub genes. The interactions of TF regulators with the hub genes, which have 40 nodes and 48 edges, are presented in Figure 9. A total of 35 TFs, like Nuclear Factor Kappa B Subunit 1 (NFKB1) and Forkhead box C1 (FOXC1) are found in TF-gene interaction networks.

### 3.5 Identification of candidate drugs

The analysis of protein-drug interactions can be helpful in the discovery of new drugs (37). In order to better understand the role of proteins and to discover potential drugs, protein-drug interactions are essential. Using EnrichR, five possible drug molecules were then predicted on the basis of transcriptome features extracted from the DSigDB database. The potential medications in the DSigDB database for the hub genes are shown in Table 4, including etoposide, 8-azaguanine, menaquinone, emetine and N-acetyl-L-cysteine (NAC). These medications have potential as therapeutic agents for HBV and TB.

**Table 4:** Potential drugs for HBV and TB.

Name	*p-*value	Chemical formula	Structure
Etoposide	1.398 × 10^−10^	C_29_H_32_O_13_	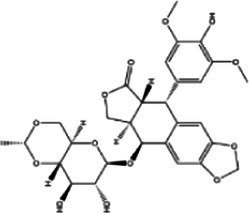
8-azaguanine	2.064 × 10^−9^	C_4_H_4_N_6_O	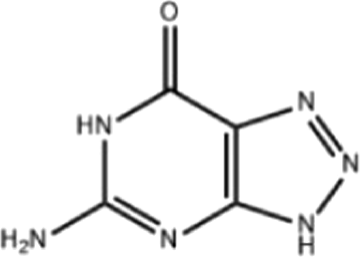
Menaquinone	4.371 × 10^−9^	C_61_H_88_O_2_	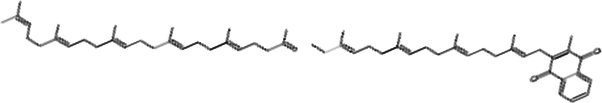
Emetine	1.072 × 10^−8^	C_29_H_40_N_2_O_4_	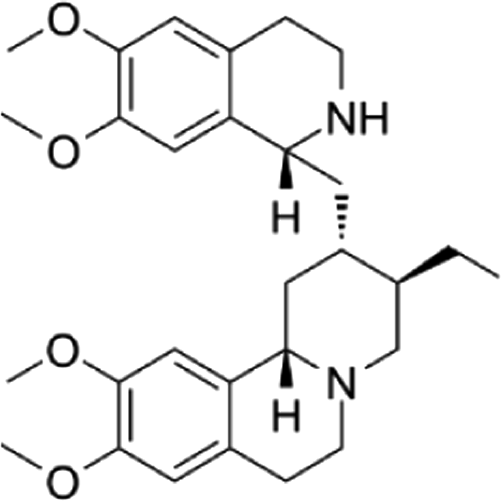
N-acetyl-L-cysteine	2.151 × 10^−3^	C_5_H_9_NO_3_S	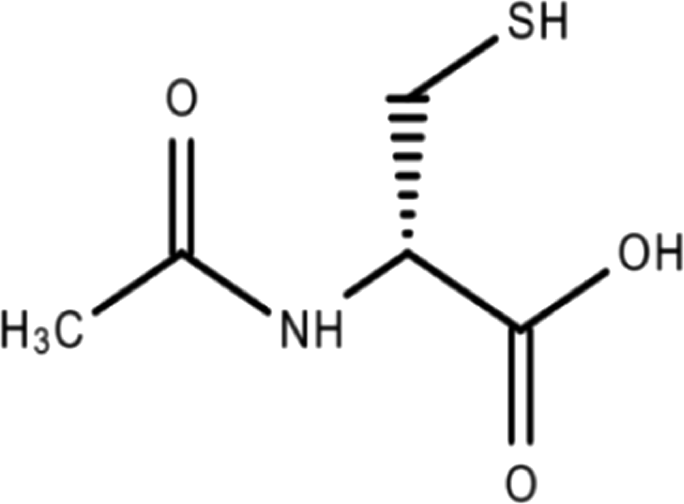

## 4 Discussion

The morbidity and mortality associated with liver diseases resulting from HBV and TB infection are considerable (38, 39). Concurrently, the aetiology of both conditions is linked to the body’s immune response (3, 40). Conversely, some studies have indicated that the chronicity of both HBV and TB infection are primarily attributable to the attenuation of the host’s immune response, the emergence of immune tolerance, and the mutation of viral molecules and the secretion of related molecules, which facilitate evasion of the body’s immune response (3, 38, 40, 41). To date, no drug has been found to be effective in preventing liver damage caused by TB drugs in HBV patients infected with TB (42). Therefore, we used bioinformatics methods to investigate the link between HBV infection and TB to find some potential drugs.

The application of enrichment analysis to gene ontologies and pathways enables the elucidation of the function and regulatory mechanisms of genes in diverse physiological states. In this study, we initially identified 35 common DEGs through differential analysis of gene expression abundance in transcriptional profiles and Venn diagram analysis. This suggests a certain degree of correlation and resemblance between HBV infection and TB in pathogenesis. Subsequently, we performed a functional enrichment analysis on the common DEGs. The results of this analysis demonstrated that these common DEGs were primarily involved in immune-related pathways and cellular metabolic regulation. The central role of aberrant activation of immune checkpoints has been demonstrated in HBV and TB infection. HBV has been observed to upregulate programmed cell death (PD-1), cytotoxic T lymphocyte antigen-4 (CTLA-4) and T cell immunoglobulin domain and mucin domain-3 (TIM-3), leading to T cell depletion (43–45). Concurrently, TB has been observed to impede T cell functionality through the PD-1/IL-10 axis (46–48). It has been demonstrated that both HBV and *Mtb* are capable of evading the host immune system by disrupting IFN signaling. Specifically, HBV has been shown to inhibit IFN production via non-structural proteins, while *Mtb* has been observed to induce a deleterious IFN response and to inhibit macrophage activation, thereby weakening the intrinsic immunity of the hepatocyte and IFN-γ-mediated antimicrobial function (4, 49, 50). Studies have shown that both HBV and TB exhibit common cellular metabolic regulation in their pathogenesis (51, 52). It has been demonstrated that *Mtb* subverts host immunity by hijacking glycolysis as evidenced by the Warburg effect and lipid metabolism. This results in the disruption of mitochondrial function and the inhibition of pro-inflammatory cytokines, such as IL-1β, to evade immune clearance (53, 54). And HBV has been observed to exert a comparable effect on the metabolic networks of the host, thereby promoting viral replication through glycolysis, lipid accumulation, and mitochondrial dysfunction (55–57).

Shared DEGs were used to construct a PPI network in which hub genes represent key regulators in the pathogenic processes of both HBV and TB. The four hub genes identified—CCL2, CD69, EGR2, and CCL20—are functionally associated with immune modulation and inflammatory responses. CCL2 is a well-known inflammatory chemokine that facilitates the migration of monocytes, memory T cells, macrophages, and natural killer (NK) cells to inflamed tissues (58). In HBV infection, the virus manipulates inflammatory signaling to support persistent replication and establish an immunosuppressive microenvironment. HBV has been shown to upregulate CCL2 expression through the activation of the TGF-β/miR-34a/CCL2 axis and by inducing mutations in the CCL2 promoter via activation of the cytidine deaminase family (59, 60). This promotes the recruitment of tumor-associated macrophages (TAMs), myeloid-derived suppressor cells (MDSCs), and regulatory T cells (Tregs), thereby dampening cytotoxic CD8^+^ T cell responses. In TB, elevated CCL2 expression is associated with disease severity and is thought to contribute to leukocyte recruitment and granuloma formation during pulmonary infection (61). CD69, a C-type lectin receptor, is one of the earliest markers of lymphocyte activation (62). In chronic HBV infection, CD69 expression is often downregulated, correlating with reduced T cell activation and antiviral function, which can be partially restored with antiviral therapy (63). In the context of TB, persistent CD69 expression on *Mtb*-stimulated T cells has been linked to impaired host defense and disease progression, potentially due to sustained immune activation and exhaustion (64). EGR2 is a transcription factor involved in the regulation of immune cell development and function. It plays a critical role in chronic HBV infection by mediating immune evasion, particularly through the regulation of Fas ligand (FasL) expression in hepatocytes in response to HBx protein (65). In TB, EGR2 is upregulated via the IFN-I-IFNGR1 signaling axis, contributing to the suppression of macrophage activation and increasing susceptibility to *Mtb* infection (66, 67). CCL20, another chemokine identified among the hub genes, exerts chemotactic effects on lymphocytes and dendritic cells through its receptor CCR6 (68). In HBV, CCL20^+^ Th17 cells are elevated and contribute to liver inflammation and fibrosis (69). In TB, upregulation of CCL20 has been observed in response to *Mtb* antigens, mediated by TNF-α/IFN-γ and the MAPK/NF-κB pathways, suggesting a role in immune cell recruitment during active infection (70). Although these findings provide plausible mechanistic insights, further experimental validation through *in vitro* or *in vivo* studies is necessary to confirm the specific roles of these genes in the co-infection context.

To better understand the pathological basis of these disease states, transcription factors that act as upstream regulators of these pivotal genes have also been identified, such as NFKB1 and FOXC1. NFKB1 is a repressor of inflammation, ageing, and cancer (71). NF-κB can inhibit HBV replication via myeloid differentiation primary response protein 88 (MyD88), a key molecule in the innate immune response signaling cascade (72). Active inflammation in response to NF-κB expression may play a key role in promoting immune clearance of HBV (73). NFKB1 is up-regulated during TB infection, and it controls the transcription of genes involved in both the pro-inflammatory and anti-apoptotic responses. *Mtb* inhibits macrophage apoptosis early in the course of infection by upregulating the NF-KB signaling pathway (39). The FOXC1 transcription factor is involved in normal embryonic development and regulates organ function (74). In HBV infection, HBV X protein significantly upregulates FOXC1 expression and transactivates its promoter activity through the Extracellular Signal-Regulated Kinase/CAMP Response Element Binding Protein (ERK/CREB) signaling pathway (75). For TB, FOXC1 is critical for mesenchymal lineage specification and organ development (76). Mesenchymal stem cells (MSCs), as a new class of phagocytes and immune cells, not only provide drug-resistant and immune-privileged ecological niches for dormant *Mtb*, but also have the ability to limit *Mtb* growth to a certain extent, which may be involved in the development of TB (77). Thus, FOXC1 could indirectly influence TB development through the regulation of MSCs. Although many previous studies have suggested that these TFs may have potential therapeutic effects, the results of these analyses require further experiments to confirm their validity and authenticity.

A series of candidate drugs targeting the DEGs were identified using the DSigDB database, including etoposide, 8-azaguanine, menaquinone, emetine, and N-acetyl-L-cysteine. Although these agents are not conventional first-line therapies for TB or HBV infection, accumulating evidence supports their potential antiviral or antibacterial effects. Etoposide, a topoisomerase II inhibitor primarily used in cancer therapy (78), has shown promise in managing severe virus-associated immune responses. For instance, etoposide was effective in a patient with virus-associated hemophagocytic syndrome (VAHS) secondary to HBV who failed to respond to standard immunosuppressive treatments (79). Moreover, etoposide combined with trichostatin A (a histone deacetylase inhibitor) overcame chemotherapy resistance in HBV-related hepatocellular carcinoma (HCC) cells via ERK inhibition and p53 activation (80). Etoposide has also been applied in TB-associated hemophagocytic lymphohistiocytosis (HLH) unresponsive to conventional anti-TB therapy (81), and recent studies indicate its ability to inhibit influenza virus replication by promoting PA subunit degradation (79). Clinical trials are currently evaluating etoposide’s use in managing COVID-19-induced hyperinflammation (80), suggesting a broader antiviral potential. 8-azaguanine, an immunomodulatory purine analog, has been shown to enhance natural killer (NK) cell cytotoxicity and exhibits antitumor and potential antiviral properties (82). It may exert its antibacterial effects through oxidative stress-related mechanisms, particularly in thiol-deficient bacteria (83). Menaquinone (vitamin K2), beyond its cardiovascular benefits (84), has gained attention in infectious disease research. Derivatives such as selenium-substituted menaquinones demonstrated strong anti-mycobacterial activity and the potential to overcome multidrug resistance in TB (85). Menaquinone biosynthesis enzymes, such as MenJ, are critical for mycobacterial survival in host cells, highlighting them as promising drug targets (86). Additionally, menaquinone has been implicated in preventing HBV progression to HCC by modulating apoptosis pathways (87, 88). Emetine, a natural alkaloid, inhibits ribosomal and mitochondrial protein synthesis and interferes with DNA/RNA activity (89, 90). It reduces cytokine production and demonstrates direct inhibitory effects on intracellular *Mycobacterium tuberculosis* by mimicking cytotoxic CD8^+^ T-cell responses (89, 91). N-acetyl-L-cysteine is a mucolytic and antioxidant compound widely used in respiratory and hepatic conditions. *In vitro*, NAC significantly reduced HBV DNA levels (92), and clinical studies have shown its benefits in HBV-related acute-on-chronic liver failure through antioxidant and anti-inflammatory effects (93). In TB, NAC limits infection by modulating host oxidative responses and inhibiting IL-6 production via MAPK/NF-κB signaling (94, 95). While these drugs exhibit promising antiviral or antibacterial properties, it is crucial to address their potential toxicity and tolerability, especially when used in combination therapy. Strategies to improve safety include dose optimization, close monitoring of immune and organ function, and combining agents such as immunomodulators or antioxidants (96, 97). Nevertheless, these approaches require further *in vivo* and *in vitro* validation to confirm efficacy and safety. Therefore, our findings should be interpreted as preliminary hypotheses, offering direction for future translational research on repurposing these compounds for HBV and TB treatment.

This research fills a critical gap in understanding the shared molecular mechanisms between HBV and TB, particularly by identifying common differentially expressed genes, enriched pathways, and potential therapeutic targets. While previous studies have largely focused on the immune response induced by a single pathogen, our study provides a systematic bioinformatics analysis of transcriptomic data from both diseases, offering a comprehensive view of their overlapping molecular signatures (38, 40). The hub genes identified in this study—such as CCL2, CD69, EGR2, and CCL20—may serve as candidate biomarkers indicating shared inflammatory or immune regulatory processes in HBV and TB (62, 98). For example, CCL2 and CCL20 are critical mediators of inflammatory cell chemotaxis, and EGR2 is involved in immune regulation and apoptosis (99). Targeting these genes may offer a rational approach for designing anti-inflammatory or immunomodulatory therapies relevant to both diseases (78). Furthermore, the drug-gene interaction analysis revealed several potential therapeutic compounds that may act on these shared targets. These findings not only offer new directions for drug repurposing and development, but also help bridge mechanistic insights into clinical applications by identifying drugs that may benefit patients with overlapping inflammatory or immune profiles across HBV and TB infections.

The identification of these genes provides valuable insights into potential therapeutic targets for both HBV infection and TB. However, several limitations should be acknowledged. First, the study relies on publicly available transcriptomic datasets that lack comprehensive clinical information, which may limit the interpretation of findings in a real-world clinical context. Second, our approach may have overlooked other important genes or signaling pathways not captured by the selected datasets or analytical criteria. Third, the absence of experimental validation using cellular or animal models limits the ability to confirm the functional roles of the identified targets. Additionally, potential methodological biases inherent in computational analyses may affect the reproducibility of results. To address these limitations, future studies will focus on incorporating multi-omics data, such as proteomics, metabolomics, and epigenetic profiles, to provide a more comprehensive view of the underlying molecular mechanisms. Experimental validation using *in vitro* and *in vivo* models will be performed to assess the biological functions and therapeutic potential of the identified targets. Moreover, efforts will be made to collect and integrate detailed patient clinical data to enable correlation analyses between gene expression changes and clinical outcomes, and to facilitate the identification of prognostic biomarkers with translational value.

## 5 Conclusion

In order to gain insight into the link between HBV infection and TB, we employed Bioinformatics and Systems Biology to identify common pathways and chemical biomarkers for HBV and TB infection. A total of 35 common DEGs for HBV and TB infection were identified through bioinformatics tools. GO and signaling pathway enrichment analysis revealed significant enrichment in immune-related pathways. Furthermore, the top four pivotal genes including *CCL2, CD69, EGR2* and *CCL20* were extracted through a PPI analysis of the DEGs, which may serve as biomarkers for HBV infection and TB treatment. We also investigated the relationship between genes and TFs like NFKB1 and FOXC1 to gain insight into potential therapeutic advances for both diseases. In addition, we have proposed a number of potential drugs, etoposide, 8-azaguanine, menaquinone, emetine and N-acetyl-L-cysteine, for the treatment of HBV infection and TB. The implications for potential new drugs of this study open up new possibilities for the treatment of HBV and TB.

## Data Availability

The datasets analyzed during the current study are available in the GEO database, https://www.ncbi.nlm.nih.gov/geo/query/acc.cgi?acc=GSE83148 and https://www.ncbi.nlm.nih.gov/geo/query/acc.cgi?acc=GSE126614.

## Footnotes

^1^ www.ncbi.nlm.nih.gov/geo/geo2r/

^2^ http://jvenn.toulouse.inra.fr/app/example.html

^3^ http://apps.cytoscape.org/apps/cytohubba

^4^ http://www.networkanalyst.ca
