# Supplementary material for: Investigation of the relationship between chronic hepatitis B and tuberculosis using bioinformatics and systems biology approaches
Source: Front Med (Lausanne). 2025 Jun 12;12:1519216. doi: 10.3389/fmed.2025.1519216 (PMC12198134; doi:10.3389/fmed.2025.1519216)
Supplement: Supplementary file 1 [file Table_1.docx]

**Supplementary Table 1.** Statistics of three clinical indicators and inflammation grades of 122 cases of hepatitis classified by age and gender.

|  |  | **Sex** |  | **Age** |
| --- | --- | --- | --- | --- |
|  |  | male | female |  |
| **Total** | 122* | 80 | 36 | 20-79 |
| **Age** |  |  |  |  |
| ≤40 | 54 | 36 | 18 | 20-40 |
| >40 | 53 | 38 | 15 | 41-79 |
| **G^#^** |  |  |  |  |
| G0 | 34 | 23 | 10 | 28-79 |
| G1 | 33 | 22 | 8 | 23-69 |
| G2 | 31 | 15 | 15 | 29-71 |
| G3 | 15 | 11 | 3 | 20-66 |
| G4 | 6 | 6 | 0 | 32-71 |
| **ALT** |  |  |  |  |
| ≤40 | 38 | 23 | 15 | 23-71 |
| >40 | 67 | 48 | 19 | 20-79 |
| **AST** |  |  |  |  |
| ≤35 | 42 | 27 | 15 | 23-71 |
| >35 | 63 | 44 | 19 | 20-79 |
| **HBV-DNA** |  |  |  |  |
| ≤10E6 | 36 | 23 | 12 | 27-71 |
| >10E6 | 54 | 37 | 17 | 20-69 |

*There were some missing data from certain samples.

**^#^**G0 and G1 were considered as mild inflammation and G2-4 as moderate or severe inflammation.
